# Factors affecting uptake of ≥ 3 doses of Sulfadoxine-Pyrimethamine for malaria prevention in pregnancy in selected health facilities, Arusha region, Tanzania

**DOI:** 10.1186/s12884-019-2592-0

**Published:** 2019-11-27

**Authors:** Witness Mkalukwatage Mchwampaka, Donath Tarimo, Frank Chacky, Ahmed Mohamed, Rogath Kishimba, Angela Samwel

**Affiliations:** 1 Mount Meru Regional Referral Hospital (MMRRH), Preventive Section, P.O. Box 3010, Arusha, Tanzania; 2 Muhimbili University of Health and Allied Sciences (MUHAS), Dar es Salaam, Tanzania; 3 Tanzania National Malaria Control Programme (NMCP), Dar es Salaam, Tanzania; 4 Tanzania Field Epidemiology and Laboratory Training Programme (TFELTP), Dar es Salaam, Tanzania; 5 Ministry of Health, Community Development, Gender, Elderly and Children (MoHCDGEC), Dar es Salaam, Tanzania

**Keywords:** IPTp-SP uptake, Malaria prevention, Tanzania

## Abstract

**Background:**

Tanzania adopted the revised World Health Organization policy in 2013 recommending a minimum of ≥3 doses of Intermittent Preventive Treatment during pregnancy with Sulfadoxine-Pyrimethamine (IPTp-SP) to protect against malaria. A study in Tanzania in 2014 reported low (9%) uptake. We investigated health workers knowledge about IPTp-SP and factors that influenced uptake of > 3 doses of IPTp-SP among pregnant women.

**Methods:**

We conducted a cross-sectional study in 2017 among post-delivery women and health care workers from nine randomly-selected public health facilities in three Districts of Arusha Region. Probability proportional to size methodology was used to determine number of participants per facility. We used a structured questionnaire to collect socio-demographic and obstetric data, information on doses of SP received, and knowledge of SP for IPTp. Health care workers were interviewed about their knowledge for IPTp- SP and challenges encountered in its uptake and use.

**Results:**

We interviewed 556 persons (median age 26 years, range 16–42 years) with the response rate of 99.3%. Of these, 484 (87.1%) had > 3 Antenatal Care (ANC) visits. A total of 402 (72.3%) were multigravida with 362 (65.1%) having given birth at least once. Of the 556 participants, 219 (39.4%) made their first ANC booking at < 17 weeks of pregnancy and 269 (48.4%) had received > 3 doses of SP-IPTp. Factors associated with uptake of > 3 doses of IPTp-SP included having secondary or higher education [Adjusted Odds Ratio (AOR) =1.6, 95%CI 1.1–2.4], attending ≥4 ANC visits [AOR = 3.1, 95%CI 2.1–4.6], having first antenatal booking at < 17 weeks [AOR = 1.8, 95%CI 1.4–2.3], and adequate knowledge on IPTp-SP [AOR = 2.7, 95%CI 1.9–3.9]. Among 36 health care workers interviewed, 29(80.6%) had adequate knowledge about IPTp-SP. SP was available in seven (87.5%) of the visited health facilities and was administered under Direct Observed Therapy (DOT) in six (75%) facilities. Health care workers reported that stock outs of SP was a challenge.

**Conclusions:**

Fewer than half of the women interviewed reported uptake of > 3 doses of IPTp-SP. That is below the Tanzania national target of 80%. Making > 4 ANC visits, having secondary or higher education, making an early first ANC visit and having adequate knowledge on IPTp-SP promoted uptake of > 3 doses. Further qualitative studies are needed to explore factors that might contribute to low uptake of SP.

## Background

Malaria infection during pregnancy is caused chiefly by *Plasmodium falciparum*, the most common malaria species in Africa [1]. Approximately fifty million women become pregnant in malaria-endemic areas each year globally, half of whom are in sub-Saharan Africa, where malaria transmission is intense and perennial [2–4]. An estimated 10,000 women and 200,000 of their infants die each year as a result of malaria infection during pregnancy, and half of these are due to severe malarial anaemia in mothers and low birth weight new-borns [5].

Recent trials in Sub-Saharan Africa, including Tanzania, have shown that uptake of ≥3 doses of Intermittent Preventive Treatment in pregnancy with Sulfadoxine Pyrimethamine (IPTp-SP) is superior to the previously recommended ≥2 doses. IPTp-SP should be delivered at each scheduled Antenatal Care (ANC) visit (except during the first trimester) and doses should be given at least 1 month apart [6, 7]. The increased doses are associated with less placenta malaria parasitaemia, higher birth weight, and increased protection of pregnant women against malaria infection [8–10]. As a result the World health Organization (WHO) now recommends use of ≥3 doses of IPTp-SP in the 2nd and 3rd trimesters of pregnancy [11].

Since the adoption of this recommendation in 2013 in Tanzania, few studies have focused on SP uptake and determinants of ≥3 doses of IPTp-SP. A study in Mozambique revealed uptake of ≥3 doses of SP to be low (43.6%) [12], while a study in Tanzania revealed even lower uptake (9%) [10]. Facility, provider, and client factors have been shown to be associated with uptake of ≥2 doses of SP for IPTp in Sub-Saharan Africa [13, 14]. However, it is not known if the same factors are also associated with the uptake of uptake of ≥3 doses regimen. We investigated individual, health provider, and facility factors that influence uptake of ≥3 doses of SP for IPTp.

## Methods

### Study site

Arusha region is in Northern Tanzania on the Kenyan border, encompassing savannah and part of the Great Rift Valley. It has a very low prevalence of malaria (< 1%) [15**]**. The Region had a total population of 1.7 million in 2012, and has an area of 14,500 m^2^. Arusha was selected for the study as it was among the first three regions in which health care workers in different health facilities levels were trained on the new policy of administering the minimum of three doses of IPTp-SP during pregnancy.

### Study design

A health facility-based cross-sectional study was conducted in nine selected Reproductive and Child Health (RCH) clinics of public health facilities from Arusha City, Meru, and Monduli councils. Public facilities were selected as they provide free delivery services, which result in most women in the region using them for deliveries.

### Study population

The study population included post-delivery women residing in Arusha region. We also included health care workers providing RCH services.

### Inclusion criteria

Any post-delivery woman in the postnatal ward in selected health facilities and had ANC card with her and any health care worker who was providing RCH services at RCH clinics in the selected study facilities were included. The data were collected for the period between April–May 2017.

### Exclusion criteria

Any post-delivery woman in whom SP use was contraindicated (known to be allergic to sulphur, or HIV-infected and therefore using cotrimoxazole prophylaxis) or who had impaired communications or did not consent to be interviewed was excluded. Any health care worker who had impaired communications or did not consent to be interviewed was excluded from the study.

### Sample size and assumptions

The sample size was obtained by using the general formula for calculating sample sizes for epidemiological studies [16].

Sample size $$ \left(\mathrm{n}\right)=\frac{{\mathrm{Z}}^2\mathrm{P}\ \left(100-\mathrm{P}\right)}{{\hbox{\pounds}}^2} $$

Where

n=Sample size required

z= Standard normal deviation corresponding to 95% confidence interval which (1.96)

P= Proportional of pregnant women who received ≥ 3-doses of IPTp-SP (estimated at 9 % [10])

£= Margin of error (estimated at 2.5%)

A small margin of error was used due to the low rate of pregnant women receiving ≥3-doses of IPTp-SP. The sample size was adjusted for non-response rate of 10%, resulting in a total of 560 post-delivery women to be included. In addition, a total number of 36 health care workers were interviewed.

### Sampling procedure

We used a multistage sampling technique to select participants. In the first, stage, three districts were selected randomly from seven Districts Council of Arusha Region. In the second stage, nine public health facilities were selected from the listed Districts, including a District hospital and two health centres. We purposefully included district hospitals since they serve the largest number of ANC clients. The same criterion was used to identify health centres to be involved in the study. In the third stage, at the selected health facilities, convenience sampling was used to recruit participants for interviews. For health care workers, six workers (at the hospital level) and three health workers (at health centre level) from RCH were selected randomly by lottery.

### Data collection for post-delivery women

Consenting post-delivery women at selected health facilities who met inclusion criteria were interviewed using a standardized structured questionnaire designed in English and translated into Swahili (Additional file 1: Questionnaire for post-delivery women). Data collected included sociodemographic, individual obstetric characteristics, and knowledge about IPTp-SP. A total of nine questions were used to assess knowledge. Providing a correct response to at least six questions was categorized as adequate knowledge. ANC cards were used to cross-check the gestational age (GA) attained, number of ANC visits, timing of ANC visits, number of IPTp-SP doses received, and GA at first ANC visit. In addition, they were used to exclude person using cotrimoxazole for prevention of Mother to Child Transmission (PMTCT), or those contraindicated to SP (those who were allergic to sulphur).

### Health care workers

Consenting health care workers at selected health facilities were interviewed using a structured questionnaire designed in English and translated in Swahili (Additional file 2: Questionnaire for health staffs) at selected health facilities. Data collected included sociodemographic, knowledge of the new regimen, and challenge with its administration. A total of 12 questions were used to assess knowledge. Providing a correct response to at least eight questions was categorized as adequate knowledge.

### Observation checklist

An observation checklist (Additional file 3: Observation checklist tool) was used to collect information on the presence of IPTp-SP health education materials such as posters and leaflets or health talks provided to patients before the start of the clinics, availability of water and cups for administering SP as Directly Observed Therapy (DOT), and availability of SP at the RCH clinic on the day of visit.

### Validity of data collection tools

The validity of all questionnaires was assessed through several processes. Firstly, we used a “Back to Back” translation method where two teams were involved; the first team formulated the questionnaires in English language then translated them into Swahili language. The second team translated the Swahili questionnaires back to English. The two teams sat together and reviewed the two English versions identifying any distortion in the meaning then revised and approved the Swahili version. Secondly, the questionnaires, both English and Swahili versions received expert review during ethical clearance process and approved for data collection. Thirdly, the approved Swahili versions of the questionnaires were pretested in the field prior to conducting study to ensure clarity and flow of questionnaire and revised accordingly. Lastly, during analysis, we checked correlation of results from related questions to assess consistency in the responses.

### Data analysis

Data were entered, cleaned, and analysed using Epi-Info version 3.5.1. Frequencies were used to summarize categorical variables and determine the proportion of post-delivery women who received three or more doses of SP for IPTp. Continuous variables were summarized by using measure of central tendencies (mean and median) and dispersion (range, standard deviation). The outcome/dependent variable (uptake of > 3 doses of SP for IPTp) was tested for association with predictors/independent variables; statistical significance was assessed by using the Chi-squared test, a *p* value of < 0.05 was set as a level of significance.

Multiple logistic regression analysis was performed to identify the significant predictors influencing uptake of > 3 doses of SP for IPTp while controlling for potential confounders. All variables with *p*-values < 0.25 in bivariate analysis were added into the multiple variable logistic regression model.

## Results

### Background characteristics of respondents

Of the 560 post-delivery women targeted, 556 (99.3%) were interviewed. Arusha City contributed 226 (40.6%). The median age of the interviewed post-delivery women was 26 years, (range, 16–42 years). Of the 556 respondents, 484 (87.1%) had made ≥3 ANC visits before delivery and 219 (39.4%) booked their first ANC visit before 17 weeks of gestation (Table 1).

**Table 1:** Demographic characteristics of post-delivery women

Variable	Frequency (n)	Percentage (%)
Age group (years)		
< 20	49	8.8
20–35	456	82
36 + yrs	51	9.2
Marital status		
Married	460	82.7
Single/widow	77	13.9
Cohabiting	19	3.4
Education level		
No formal education	42	7.6
Primary education	273	49.1
Secondary education	200	35.9
College/University	41	7.4
Occupation		
Farmer	101	18.2
Housewife	154	27.7
Petty business/self employed	232	41.7
Employee (Private/public)	69	12.4
Number of ANC visits		
< 4	243	43.7
> 4	313	
Gravidity		
Prima gravida	154	27.7
Multigravida	402	72.3
Parity		
0	194	34.9
> 1	362	65.1
GA at first ANC visit		
< 17 weeks	219	39.4
> 17 weeks	337	60.6
Knowledge about IPTp-SP		
Adequate	273	49.1
Low	283	50.9
Walking time to ANC facility in (min)		
< 30	342	61.5
> 30	214	38.5

### Proportion of post-delivery women with uptake of > 3 doses of IPTp-SP

In total, 269 (48.4%) women received ≥3 doses of SP for IPTp, 150 (27%) received 2 doses, 110 (19.7%) received one dose, and 27 (4.9%) did not receive IPTp-SP despite attending at ANC at least twice. Of the interviewed women, 284 (51.1%) reported not receiving SP at ANC in at least one of their routine ANC visits. Of these, 184 (65.8%) reported it was due to unavailability of SP at the health facility. Of the 529 (95.1%) women who received at least one dose of SP, 149 (28.1%) they reported that they did not swallow SP in front of the health care providers due to; absence of clean water or cups at the ANC clinics 74 (50%), and being hungry 20 (13.4%). Others did not mention the reasons but were given medication to take at home.

### Factors influencing uptake of > 3 doses of IPTp-SP

After adjusting for confounding variables, factors that remained significantly associated with the uptake of ≥3 doses of SP for IPTp at the multivariate level included having secondary or higher education [Adjusted Odds Ratio (AOR) = 1.6, 95%CI 1.1–2.4], having visited an ANC at least four times [AOR = 3.1, 95%CI, 2.1–4.6], having their first ANC visit at < 17 gestation age (GA) [AOR = 1.8, 95%CI 1.4–2.3], having adequate knowledge of IPTp-SP [AOR = 2.7, 95%CI, 1.8–3.8], and having delivered at least once previously [AOR = 1.7, 95%CI 1.2–2.6] (Table 2).

**Table 2:** Factors associated with uptake of ≥3 doses of IPTp-SP by post-delivery women

Variable	Crude OR (95%CI) Adjusted OR (95%CI)	
Age group (yrs.)		
> 20	1.8 (1.15–2.89)	1.1 (0.60–1.83)
< 20	Ref	Ref
Education level		
Secondary/college	1.9 (1.42–2.81)	1.6 (1.11–2.42)**
Non/primary	Ref	Ref
Occupation		
Employed/self employed	1.6 (1.15–2.26)	0.9 (0.59–1.28)
Farmer/housewife	Ref	Ref
Number of ANC visit		
> 4	3.5 (2.48–5.04)	3.1 (2.12–4.62) **
< 4	Ref	Ref
Gravidity		
Prima gravida	1.4 (0.97–2.05)	1.2 (0.82–1.68) Ref
Multigravida	Ref	
Parity		
+ 1	1.6 (1.09–2.21)	1.7 (1.22–2.68) **
0	Ref	Ref
GA at first ANC booking (wks.)		
< 17	1.6 (1.15–2.29)	1.8 (1.42–2.30) **
> 17	Ref	Ref
Knowledge of P. women on IPTp-SP		
Adequate	3.1 (2.17–4.33)	2.7 (1.85–3.89) **
Low	Ref	Ref
Distance (walking time) to HF		
< 30 min	1.5 (1.07–2.14)	1.3 (0.88–1.89)
> 30 min	Ref	Ref

^*^Statistically Significant (*p* = < 0.05)

### Health worker interviews

#### Demographics characteristics

A total of 36 health care workers were interviewed in nine health facilities. The mean age was 39.5 years (range, 19–60 years), and 35 (97.2%) were female (Table 3). In total, 29 (80.6%) had adequate knowledge of IPTp-SP. All the interviewed health workers reported administering SP at the ANC when it is was available and directing women to swallow it in front of health care provider.

**Table 3:** Sociodemographic characteristics of Health Care Workers (HCW)

Variable	No of Health workers *n* = 36	Parentage
District		
Arusha City	12	33.3
Meru	12	33.3
Monduli	12	33.3
Level of Facility		
Hospital	18	50
Health centre	18	50
Age in years		
< 35	11	30.6
> 35	25	69.4
Sex		
Female	35	97.2
Male	1	2.8
Title		
Public health nurse	3	8.3
Enrolled nurse	5	13.9
Nurse assistant	6	16.7
Nurse Midwife	13	36.1
Registered nurse	9	25.0

Eight (88.9%) of the nine selected health facilities underwent observation of health care workers using the checklist.

It was observed that health education regarding malaria and specifically SP for IPTp was given before the start of services. SP was available at seven (87.5%) health facilities at the ANC; however, only three (37.5%) had IPTp national protocols and two (25.0%) had IPTp training manuals (Table 4). Five (62.5%) health facilities had posters of IPTp or Malaria in Pregnancy (MIP) on their walls; however, some of these messages were in English and more scientific. Free, clean, and safe water was observed in seven (87.5%) facilities; however, DOT was observed in only six (75.0%). Forms for reporting adverse events associated with SP were available only in four (50.0%) of the visited health facilities.

**Table 4:** Observation of practice of health workers on administering SP for IPTp

Variable	No.of facilities	Percentage (%)
Health talk given at ANC on day of visit	8	100
Health talk given that day included malaria in pregnancy	8	100
Health talk given that day included IPTp	8	100
Presence of request forms for ANC medicines including SP	3	37.5
Presence of posters of IPTp/MIP on the wall	5	62.5
Presence of ANC Report Book for daily summaries	8	100
SP given is recorded in ANC report Book for daily summaries	8	100
SP given is recorded in ANC cards of clients	8	100
SP available at ANC	7	87.5
Practice of DOT observed	6	75
Presence of Adverse Effects forms for SP	4	50
Presence of free, clean, safe water for DOT	7	87.5
Presence of safe, clean water for sale for DOT	0	0
Availability of IPTp National protocol	3	37.5
Availability of IPTp training manual	2	25

#### The stated challenges of administering SP for IPTp

Among the 36 interviewed health workers, 25 (69.4%) reported running out of SP at the ANC clinic at some point during the past 3 months. Of these, 12 (48%) reported that it occurred twice in the past 3 months, and nine (36%) reported that it occurred once; the other four (16%) could not recall. Pregnant women who missed medication on those days were asked to go and buy SP outside. Thirty-three (91.7%) reported that their clinics supplied clean and safe water for pregnant women to swallow SP in front of the health care provider, and two (8.3%) reported giving the medicine to pregnant women to swallow at home.

## Discussion

Four years after adopting new recommendations nationwide to provide pregnant women with ≥3 doses of SP for IPTp, actual uptake in the Arusha region of Tanzania was low. Fewer than half of the women interviewed received at least three doses. Independent factors that significantly influenced uptake of ≥3 doses of IPTp-SP included having visited the ANC four or more times, having booked their first ANC visit < 17 weeks of gestation, having adequate knowledge on IPTp-SP, having secondary or higher education, and having delivered at least once previously. Despite the fact that a ≥ 3 dose of SP for IPTp is superior to two doses, it is not fully utilized. Challenges to administration, including stock outs, may be partially related to the poor uptake.

The uptake of > 3 doses of IPTp-SP in the present study is lower (48.4%) the national target of 80%. However, it is higher than the uptake of 9% reported two years earlier in Tanzania [10]. Unavailability of SP at the health facility was reported in this study, has been reported in several previous studies that focused on two-dose regimens [14, 17]. Stockouts present barriers to achieving the target and leave pregnant women unprotected from malaria. Practice of DOT in this study and other studies in Sub Saharan Africa [17] was found to be hampered by lack of knowledge among health care workers about the safety of SP on empty stomach as well as lack of availability of safe water and or cups.

Having made a first visit to an ANC early, as recommended by Tanzania Focused Antenatal Care (FANC), improved uptake of ≥3 doses of SP for IPTp. This was also observed in studies in Zimbabwe and Tanzania that focused on two- dose regimen [5, 18]. Similarly, having visited the ANC more than four times influenced uptake of > 3 of SP for IPTp. These findings are consistent with other studies conducted in Tanzania and West Africa [3, 5, 15]. This may be due to having an increased chance of receiving repetitive health education messages on ANC services, including the importance of SP, as well as increased opportunities to receive any missed doses at her multiple ANC visits.

Knowing about IPTp-SP was another independent factor associated with uptake of ≥3 doses of SP for IPTp. This was also reported in studies in Tanzania and Ghana that focused on the two-dose regimen [19, 20]. These suggest that if clients are knowledgeable of the importance of receiving SP, they are more likely to attend antenatal clinic to receive it. We also found that most women interviewed received information on IPTp from the ANC clinics during their routine visits, and nearly all held positive perceptions of IPTp with SP for preventing malaria during pregnancy. This shows that pregnant women are equipped and therefore empowered with the correct information from the health care workers, which is in line with the FANC guidelines [21]. However, other means of delivering health education messages regarding IPTp should also be taken into accounts such as using the media (radio, television), leaflets, or others methods so that even pregnant women who were not planning to attend ANC early can find out the importance of attending to receive SP for IPTp.

We found that health care workers were knowledgeable about malaria prevention in pregnancy using SP. However, this knowledge might not be transferred to pregnant women who attend ANC clinics, as more than a half of post-delivery women had low knowledge about IPTp-SP. This may result in challenges accepting the drug when these pregnant women are issued SP. A study conducted in rural Nigeria showed that, despite of the pregnant women having heard about IPTp, 44 (40.4%) women were afraid of taking drugs during pregnancy [22].

This study includes few limitations: the lack of generalizability to all health facilities as the study focused on public facilities. In addition, the questionnaire required some information from during the early stage of pregnancy that might have introduced recall bias. The ANC card was used to verify some of the key variables, which may have limited this type. Finally, although efforts were made to control for confounders at analyses stage, due to the cross-section design, unidentified confounders may still have affected the observations.

## Conclusion

The uptake of ≥3 doses of IPTp-SP in Arusha during 2017 was below the Tanzania national target of 80% coverage. In this study, the same factors that were previously shown to influence uptake of the two-dose regimen were found to influence uptake of ≥3 doses of SP for IPTp. Having made ≥4 ANC visits, having first visited the ANC at an early gestational age, having knowledge on IPTp-SP, having secondary or higher education, and having delivered at least once prior to the current delivery were significantly associated with the uptake of ≥3 or more doses of SP for IPTp. Strategies to ensure constant availability of SP tablet at the RCH clinics along with a constant supply of clean water and cups at the health facilities should be emphasized. The IPT programme should ensure provision of standardized improved Information, Education and Communication (IEC) materials that contain clear and well understood information. This information should show not only the benefit of IPT, but also the number of doses and gestational age at which pregnant women should take this dose.

## Supplementary information

**Additional file 1 **Questionnaire for post-delivery women. The tool used to interview the post-delivery woman to assess factors affecting uptake of > 3 doses of Sulfadoxine Pyrimethamine for Malaria prevention in Arusha.

**Additional file 2.** Questionnaire for health facility staffs. This tool was used to collect information from health workers working in the department of Obstetrics and Gynaecology on their demographic characteristics, knowledge on the new regime of administering the minimum of three doses during pregnancy and challenges associated with administering IPT-SP.

**Additional file 3.** Observation checklist. This tool was used to collect information on the presence of IPTp-SP, health education materials such as posters and leaflets or health talks provided to patients before the start of the clinics, availability of water and cups for administering SP as directly observed therapy (DOT), and availability of SP at the RCH clinic on the day of visit.

## Data Availability

The data used and/or analysed during the current study are available from the corresponding author on reasonable request.

## Abbreviation

ANC: Antenatal Care
CHMT: Council Health Management Team
DOT: Directly Observed Therapy
FANC: Focused Antenatal Care
HCW: Health care workers
HF: Health facility
IPT: Intermittent Preventive Treatment
IPTp: Intermittent Preventive Treatment in pregnancy
ITN: Insecticide treated net
MoHCDGEC: Ministry of Health, Community Development, Gender, Elderly and Children
PMTCT: Prevention of Mother to Child Transmission
RCH: Reproductive and child health
RCH: Reproductive and Child Health
SP: Sulfadoxine- Pyrimethamine
TDHS: Tanzania Demographic and Health Survey
THMIS: Tanzania HIV/Malaria Indicator Survey
WHO: World Health Organization
