# Supplementary material for: Factors affecting uptake of ≥ 3 doses of Sulfadoxine-Pyrimethamine for malaria prevention in pregnancy in selected health facilities, Arusha region, Tanzania
Source: BMC Pregnancy Childbirth. 2019 Nov 27;19:440. doi: 10.1186/s12884-019-2592-0 (PMC6880562; doi:10.1186/s12884-019-2592-0)
Supplement: Supplementary file 1 — Additional file 1 Questionnaire for post-delivery women. The tool used to interview the post-delivery woman to assess factors affecting uptake of > 3 doses of Sulfadoxine Pyrimethamine for Malaria prevention in Arusha. [file 12884_2019_2592_MOESM1_ESM.docx]

**SUPPLEMENTARY FILES ON A STUDY TITTLED** ‘’**Factors affecting uptake of ≥3 doses of Sulfadoxine-Pyrimethamine for malaria prevention in pregnancy in selected health facilities, Arusha region, Tanzania’’**

***Supplementary file 1:***

**Questionnaires for post-delivery women**

Date of interview…………… Time start……………

Name of interviewer………………….

**INTRODUCTION**: We are healthcare workers from Mount Meru regional referral hospital; we are here to collect information on intermittent preventive therapy for pregnant women (IPTp) use. This information is going to be used for research purpose and its outcome is expected to be used as basis of improving IPT use among pregnant women. We therefore request your participation in this interview.

**SECTION1: IDENTIFICATION AND SOCIO-DEMOGRAPHIC FACTORS**

**Instructions**: Please follow instructions under each section before interviewing the client. All the instructions are written in italics. Fill in the correct answer for questions 1 -11 in this section. For the rest of other questions (unless shown) circle the number of the corresponding response. **Note**: Do not read the responses for the client to choose the right answer. Read the question and wait for the response.

Q1.ID number--------------------

Q2.Name of the District------------------------------

Q3.Name of the facility----------------------------------------

Q4.Level of facility--------------------------------

Q5.Which year were you born? ------------- Age in years

Q6.Gestation age (GA) in weeks (***Record GA from MCH card***)

***When was your LMP---------------------------- (compute GA).Gestation age in wks.***

Q7.Gravidity :( No of pregnancy the pregnant woman has ever had including the current pregnant) ***(Confirm from the MCH card)***

Q8.Parity :( Number of times a pregnant woman has delivered)***(Confirm from the MCH card)***

Q9.Total number of routine ANC visit including the visit on the day of interview

***(Confirm on the MCH)-***

Q10.Gestation age (wks.) at first ANC visit***(record from MCH card)***

Q11.Number of SP doses given/taken*(****record from MCH card****)*

Q12.Marital Status

1. Married
2. Widow
3. Divorced
4. Single
5. Co-habiting

Q13.Highest level of education attained

1. No formal education/didn’t complete primary education
2. Completed Primary education
3. Didn’t complete secondary education
4. Complete secondary education IV
5. Completed secondary education VI
6. College/university

Q14.Occuppation which is the main source of income

1. Housewife/depend on my husband
2. Farmer
3. Petty business/self employed
4. Employee (public/private)

**SECTION 2: ANTENATAL CARE ACCESS AND KNOWLEDGE OF ANC SERVICES*(read the options and circle the no.of the mentioned response/follow instruction written in the brackets for other Questions)***

Q15.Approximate walking time in minutes from your home to the closest health facility with the ANC services........................... ***(Fill in the blanks for the mentioned response)***

16. Means of transport used to reach at the health facility with ANC services------------------------------------------------------- ***(Fill in the blanks for the mentioned response)***

Q17.How many hours do you spend on average at ANC (from the time you arrive up to the time you live)---------------------------------------- ***(Fill in the blanks for the mentioned response)***

Q18.Who advised you to seek ANC services ***(circle the mentioned responses***)

1. Self-initiative
2. Husband/Spouse
3. Relatives
4. Heard from the radio/television/read the newspapers
5. Neighbours
6. Hospital referrals

Q19.When is it recommended for a pregnant woman to start seeking ANC services?

1. Once discovered pregnant
2. At 16 weeks
3. At 20 weeks
4. >20weeks
5. When about to deliver
6. Don’t know

Q20.What is the recommended interval time (weeks) between one ANC visits and another

1. 2weeks
2. 4weeks
3. 8weeeks
4. >8weeks
5. Don’t know

**SECTION 3: KNOWLEDGE OF PREGNANT WOMEN ON IPTp AND OTHER MALARIA PREVENTION METHODS *(read options and circle the mentioned response)***

Q21. Have you ever heard of any preventive measure of malaria during pregnancy?

1. Yes
2. No **(*If No, go to (Q23*)**

Q22. Where did you hear about prevention of malaria in pregnancy? *(****Circle all that applies)***

1. Antenatal Clinic
2. Television
3. Radio
4. Leaflets
5. Relatives/friends
6. Can’t remember
7. Other (Specify)---------------------------------

Q23. What methods can you use to prevent malaria during pregnancy ***(Read the on and circle the numbers of the mentioned responses)***

1. Use of drugs (IPT) for malaria prevention
2. Use of LLIN for malaria prevention
3. Use of mosquito repellent for malaria prevention
4. Wearing of protective clothing
5. Spray insecticides inside the house
6. Drain stagnant water
7. Clear grass and bushes around the home
8. Don’t know
9. Others specify------------------------

Q24. What drug is used for malaria prevention during pregnancy at ANC clinic?

1. SP (Sulfadoxine Pyrimethamine)
2. Other mentioned antimalarial apart from SP
3. Don’t know(***if response is not 1 go to (Q33)***

Q25. What is the minimum dose of SP is required during the entire pregnancy?

1. One
2. Two
3. Three
4. > Three
5. Don’t know

Q26. How many doses of SP are recommended for a pregnant woman to take during her entire pregnancy?

1. One
2. Two
3. Three
4. Three and above
5. Don’t know

Q27. When is the recommended gestation age for the first dose of SP for IPTp

1. <12weeks
2. As early as possible after 12 weeks
3. 20-24weeks
4. 28-32weeeks
5. >32weeks
6. Don’t know

Q28. What is the time interval between one dose of SP and the next dose

1. 2weeks
2. 4weeks
3. 8weeks and above
4. Don’t know

Q29. To your knowledge up to whatperiod can SP be given during pregnancy

1. Up to 32 weeks
2. Up to 36weeks
3. Up to delivery
4. Don’t know

Q30.Where is the recommended place for SP to be swallowed

1. In front of ANC provider
2. At home
3. Anywhere in the hospital
4. Don’t know
5. Other specify-----------------------------------

Q31. During which period (GA in weeks) is SP not allowed to be given during pregnancy

1. 12weeks and below
2. 24-36weeks
3. Above 36 weeks
4. Don’t know

Q32. During pregnancy what are condition which can hinder a pregnant woman from taking SP

1. HIV positive woman who is using cotrimoxazole and or reactions such as itching
2. Vomiting
3. Diarrhoea
4. Don’t know
5. Other specify---------------------------------

**SECTION 4 PRACTICE OF DOT AND ATTITUDE OF PREGNANT WOMEN TOWARDS IPTp *(read options and circles the mentioned response)***

Q33.Did the service provider give you medicine (SP) today?

1. Yes
2. No

Q34. Have you ever been given SP by service provider during the previous ANC visits

1. Yes ***(if response is 1 go to Q36)***
2. No

Q35.If no to Q33/ 34, what were the reasons given by the service provider for not giving SP. (***after this go toQ44****)*

1. There was no medicine at health facility
2. I had no money to buy the medicine
3. This is my first ANC visit
4. Don’t know
5. Others specify--------------------------------------

Q36.If yes to question 33/ 34 how many tablets were they?

1. One
2. *Two*
3. Three
4. > three
5. Don’t remember

Q37. Did the medicine look like this one? ***(Please show a sample of SP***)

1. Yes ***(if yes go to Q39)***
2. No

Q38. If no, how did it look like? .............................................................................

Q39. Do you usually swallow SP in front of service provider at ANC?

1. Yes to all the visits
2. No to some visits
3. No to all the visits (***ifno go to q43)***

Q40.How many times did you swallow SP at ANC

1. Once
2. Twice
3. Thrice
4. Four times
5. Five times
6. Can’t remember
7. Don’t know

Q41. Were you served with free, clean water to take the medicine?

1. Yes ***(go to Q44)***
2. No

Q42.If no, how did you get the water to take the medicine?

1. Bought water at the ANC
2. Fetched water from the tap
3. Bought water at the health facility
4. Had my own water
5. Other specify------------------------------------

Q43.If no/no to some visits to Q39, what were the reasons for not swallowing SP in front of service provider

1. There was no clean water at ANC
2. There was no enough cups to use at ANC
3. I was hungry/had not eaten yet
4. There was no medicine at the facility
5. I don’t know
6. Other specify………………………………

Q44. Do you think that SP is effective in preventing malaria in pregnancy?

1. Yes ( ***If yes, go to question46***)
2. No
3. Don’t know

Q45.If no to Q44, What reasons make you think SP is not effective in IPT?

1. It has shown resistance in treating malaria
2. It is no longer used for Malaria treatment
3. It has side effect to my baby
4. Don’t know
5. Other (Specify)…………

Q46. What do you think can be the effects of malaria during pregnancy (***Circle the number of the mentioned responses)***

1. Can cause anaemia
2. Can cause death
3. Can cause spontaneous abortion
4. Can cause intrauterine foetal death
5. Can cause prematurity
6. Can cause low birth weight
7. Don’t know
8. Others specify---------------------------------

Q47.Knowledge score………………

**ROM THE ANTENATAL CARD**

| **ANC Visits** | **IPTp-SP dose** | **Gestation age (weeks)** |
| --- | --- | --- |
| First |  |  |
| Second |  |  |
| Third |  |  |
| Fourth |  |  |
| >Fifth |  |  |
