# Supplementary material for: Factors affecting uptake of ≥ 3 doses of Sulfadoxine-Pyrimethamine for malaria prevention in pregnancy in selected health facilities, Arusha region, Tanzania
Source: BMC Pregnancy Childbirth. 2019 Nov 27;19:440. doi: 10.1186/s12884-019-2592-0 (PMC6880562; doi:10.1186/s12884-019-2592-0)
Supplement: Supplementary file 2 — Additional file 2. Questionnaire for health facility staffs. This tool was used to collect information from health workers working in the department of Obstetrics and Gynaecology on their demographic characteristics, knowledge on the new regime of administering the minimum of three doses during pregnancy and challenges associated with administering IPT-SP. [file 12884_2019_2592_MOESM2_ESM.docx]

**SUPPLEMENTARY FILES ON A STUDY TITTLED** ‘’**Factors affecting uptake of ≥3 doses of Sulfadoxine-Pyrimethamine for malaria prevention in pregnancy in selected health facilities, Arusha region, Tanzania’’**

## Supplementary file 2: Questionnaire for health staffs

Date of interview…………… Time start……………….

Name of interviewer………………….

**INTRODUCTION**: We are healthcare workers from Mount Meru regional referral hospital; we are here to collect information on intermittent preventive therapy for pregnant women (IPTp) use. This information is going to be used for research purpose and its outcome is expected to be used as basis of improving IPT use among pregnant women. We therefore request your participation in this interview.

**SECTION1: IDENTIFICATION AND SOCIO-DEMOGRAPHIC FACTORS**

**Instructions**: Please follow instructions under each section before interviewing the client. All the instructions are written in italics. Fill in the correct answer for questions 1 -7 in this section. For the rest of other questions (unless shown) circle the number of the corresponding response. **Note**: Do not read the responses for the client to choose the right answer. Read the question and wait for the response.

Q1.ID number--------------------

Q2.Name of the District------------------------------

Q3.Name of the facility----------------------------------------

Q4.Level of facility------------------------------

Q5.Which year were you born------------------, Compute age in years-----------------

Q6. Sex --------------------------------------

Q7.The period (in months) you have worked in medical field---------------------------------

Q8. Staff category:

1. Nurse assistant
2. Enrolled nurse
3. Nurse Midwife
4. Public health nurse
5. Registered nurse
6. Other, specify-------------------------------

**SECTION 2; KNOWLEDGE OF ANC STAFFS ON Intermittent Preventive Therapy of malaria using Sulfadoxine-Pyrimethamine –IPTp-SP**

Q9. What drug is used for malaria prevention during pregnancy?

1. SP (Sulfadoxine Pyrimethamine)
2. Other mentioned antimalarial apart from SP
3. Don’t know(***if response is not 1 go to (Q21)***

Q10. What is the minimum dose of SP is required during the entire pregnancy?

1. One
2. Two
3. Three
4. > Three
5. Don’t know

Q11. How many doses of SP are recommended for a pregnant woman to take during her entire pregnancy?

1. One
2. Two
3. Three
4. Three and above
5. Ohers specify--------------------
6. Don’t know

Q12. When is the recommended gestation age for the first dose of SP for IPTp?

1. <12weeks
2. As early as possible after 12 weeks
3. 20-24weeks
4. 28-32weeeks
5. >32weeks
6. Others specify------------------------
7. Don’t know

Q13. What is the time interval between one dose of SP and the next dose?

1. 2weeks
2. 4weeks
3. 8weeks and above
4. Others specify-----------------
5. Don’t know

Q14. To your knowledge up to whatperiod can SP be given during pregnancy?

1. Up to 32 weeks
2. Up to 36weeks
3. Up to delivery
4. Don’t know

Q15.Where is the recommended place for SP to be swallowed?

1. In front of ANC provider
2. At home
3. Anywhere in the hospital
4. Don’t know
5. Other specify-----------------------------------

Q16. During which period (GA in weeks) is SP not allowed to be given during pregnancy

1. 12weeks and below
2. 24-36weeks
3. Above 36 weeks
4. Other specify----------------------
5. Don’t know

Q17. During pregnancy what are condition which can hinder a pregnant woman from taking SP for IPTp

1. HIV positive woman who is using cotrimoxazole and or reactions such as itching
2. Vomiting
3. Diarrhoea
4. Other specify---------------------------------
5. Don’t know

Q18. If a pregnant woman is diagnosed with malaria during routine ANC attendances, what will you do?

1. Treat the patient with SP
2. Withhold SP for some time and let the patient be treated with ALU/Quinine
3. Don’t know
4. Others specify---------------------------------

Q19.If a pregnant woman who attends NC is allergic to SP, what will you do for prevention of malaria during pregnancy?

1. Will stop giving sulphur completely
2. Will give ant allergic dugs and encourage her to continue with SP
3. Will prescribe other ant malaria for preventive
4. Don’t know
5. Other specify--------------------

Q20.If a pregnant is HIV positive and is using cotrimoxazole, what will you do for prevention of malaria during pregnancy

1. Will continue to give SP
2. Will stop giving sulphur completely
3. Will prescribe other ant malaria for preventive
4. Don’t know
5. Other specify--------------------------

**SECTION 3: PRACTICE OF IPTp –SP AT ANC AND AVAILABILITY OF SP AT THE CLINIC (*Read the response and circle the mentioned response)***

Q21. Do you administer SP for IPTp at the RCH clinic?

1. Yes all the days
2. Yes to some days
3. No to all the days***(Go to Q23)***

Q22. How is the medicine administered at your clinic? ***(Go to Qn 24)***

1. Given to the pregnant women to take home
2. We observe the pregnant women take the medicine at the clinic
3. Prescriptions are written for the pregnant women to go and collect at the pharmacy
4. Other specify---------------------------------

Q23.If no/yes to some days to Q21, what are the reasons for not administering SP at the clinic?

1. Unavailability of trained health workers to administer SP
2. Unavailability of medicine at the facility
3. Unavailability of safe water
4. Unavailability of cups/enough cups at the health facility
5. Don’t know
6. Other specify-------------------------------

Q23. Have you ever run out of the medicine for IPTp in your clinic?

1. Yes
2. No
3. Don’t know

(***If response is 2/3 go to Q26)***

Q24. (If yes) to ***Q23****,*how many times during the last three months?

1. Once
2. Twice
3. Don’t know
4. Other (Specify)…………

Q25. What happened to the IPTp programme when there was no SP?

1. Suspended till we got the medicine
2. Asked women to buy SP 3) Referred women to other Health Facilities
3. Don’t know
4. Other, specify……

Q26. Where do you normally get the supplies of medicine for IPTp from?

1. Private pharmacies
2. Medical Stores Department
3. Don’t know
4. Other, specify ………………………………….

Q27. Do you supply clean safe water for the pregnant women to take the IPTp medicine?

1. Yes
2. No

Q28.If no, how do the women get water for the medicine?

1. Buy water from the clinic
2. Bring water from outside the unit
3. They take the medicine when they go back home.
4. Other, specify…………………
