# Supplementary material for: Factors affecting uptake of ≥ 3 doses of Sulfadoxine-Pyrimethamine for malaria prevention in pregnancy in selected health facilities, Arusha region, Tanzania
Source: BMC Pregnancy Childbirth. 2019 Nov 27;19:440. doi: 10.1186/s12884-019-2592-0 (PMC6880562; doi:10.1186/s12884-019-2592-0)
Supplement: Supplementary file 3 — Additional file 3. Observation checklist. This tool was used to collect information on the presence of IPTp-SP, health education materials such as posters and leaflets or health talks provided to patients before the start of the clinics, availability of water and cups for administering SP as directly observed therapy (DOT), and availability of SP at the RCH clinic on the day of visit. [file 12884_2019_2592_MOESM3_ESM.docx]

**SUPPLEMENTARY FILES ON A STUDY TITTLED** ‘’**Factors affecting uptake of ≥3 doses of Sulfadoxine-Pyrimethamine for malaria prevention in pregnancy in selected health facilities, Arusha region, Tanzania’’**

## Supplementary file 3: Check list, observation

Date---------------------------------------------------

Name of facility -------------------------------------

District -----------------------------------------------
**Please put tick (v) on the mentioned response**

| Health talk given at ANC on day of visit | YES | NO |
| --- | --- | --- |
| Health talk given that day included malaria in pregnancy |  |  |
| Health talk given that day included IPTp |  |  |
| Presence of request forms for ANC medicines including SP |  |  |
| Presence of posters of IPTp/MIP on the wall |  |  |
| Presence of ANC Report Book for daily summaries |  |  |
| SP given is recorded in ANC report Book for daily summaries |  |  |
| SP given is recorded in ANC cards of clients |  |  |
| SP available at ANC |  |  |
| Practice of DOT observed |  |  |
| Presence of Adverse Effects forms for SP |  |  |
| Presence of free, clean, safe water for DOT |  |  |
| Presence of safe, clean water for sale for DOT |  |  |
| Availability of IPTp National protocol |  |  |
| Availability of IPTp training manual |  |  |
